# Prevalence of Attention Deficit Hyperactivity Disorder Symptoms in Children Who Were Treated at Emergency Service due to Unintentional Injury

**DOI:** 10.1155/2018/7814910

**Published:** 2018-12-12

**Authors:** Mehmet İz, Veysi Çeri

**Affiliations:** ^1^Şanlıurfa, Mehmet Akif İnan Trainig and Research Hospital Emergency Department. Şanlıurfa, Turkey; ^2^Marmara University, School of Medicine, Department of Child and Adolescent Psychiatry, Pendik/İstanbul, Turkey

## Abstract

**Aim:**

Attention Deficit Hyperactivity Disorder (ADHD) is a developmental disorder characterized by severe inattention, hyperactivity, and impulsivity. This research aims to determine the frequency of ADHD symptoms in children who were treated in emergency paediatric services due to unintentional injuries.

**Method:**

This study was carried out with children who were treated due to unintentional injuries in an Emergency Department. ADHD symptoms were evaluated using the DSM-IV-based Screening and Assessment Scale for Behavioural Disorders in Children and Adolescents.

**Results:**

The study sample consisted of 89 girls (40.1%) and 133 boys (59.9%)—a total of 222 children. The participants ranged from 5 to 18 years of age, and the mean age was found to be 11.5±3 years. According to medical evaluations, the most common diagnosis for the unintentional injuries was soft tissue trauma (41.9%). The mean ADHD and ODD (Oppositional Defiant Disorder) scores of our study sample were, respectively, 19.9±12 and 7.7±5.7. The prevalence of children with possible ADHD was as high as 81.6% (179) and, for ODD, was 62.6% (139), according to cut-off values.

**Conclusion:**

Our results pointed out very high levels of ADHD and ODD symptoms among children who were treated at emergency services for accidental injuries. Appropriately screening for ADHD in children with accidental injuries and referring them to child psychiatry units may prevent later accidents and injuries.

## 1. Introduction

Responsible for 830,000 childhood deaths every year, accidents and injuries are a major public health problem as they are one of the main causes of death and permanent disability in children and adolescents [1, 2]. Peden (2008) notes that the number of children who died from accidents and injuries is higher than the total number of children who died from various infectious diseases [3]. She also asserts that preventive practices improve the health of children and should, therefore, be included in programs developed for that purpose [3]. In preventing accidents, it is important to determine the risk factors associated with accidents and injuries, as well as the characteristics of children who are likely to suffer accidents, thus removing these risk factors [4].

Attention Deficit Hyperactivity Disorder (ADHD) is a developmental disorder characterized by severe inattention, hyperactivity, and impulsivity [5]. In community studies with different cultures, ADHD is observed in 5% of children [5, 6]. In studies conducted in Turkey, the ratio was approximately 13%, and the ratio did not change very much over the years [7].

ADHD causes loss of functioning, which has a negative impact on the academic and social lives of children [8, 9]. Besides generating deviations in social and academic development, ADHD makes children more vulnerable to accidents and injuries for various reasons, but especially because of inattentiveness and impulsivity [10]. Several studies have corroborated this point, illustrating that these children are indeed more prone to accidents and injuries [4, 11]. For example, in a study comparing children with and without ADHD, those in the ADHD group were more likely to be referred to emergency services and had a higher rate of recurrent injuries [12]. However, alongside growing evidence that ADHD makes children more susceptible to accidents, other studies suggest that these outcomes are controversial [4], that ADHD does not predispose people to accidents, and that the incidence of accidents in these children may be related to other factors [13]. Nevertheless, the sheer volume of research pointing to ADHD as a predisposition to accidents is too substantial to ignore.

It has also been suggested that ADHD may be accompanied by decreased life expectancy and increased accident and injury rates are thought to be one reason for this [8, 14]. Along with struggles maintaining attention, impulsivity, risky behaviours, difficulties in motor coordination, and problems with executive functions also make children and adolescents with ADHD more vulnerable to accidents [15]. The risk of injury is increased when ADHD and Oppositional Defiant Disorder (ODD) accompanied one another [16].

The types of injuries associated with ADHD vary with age. For example, foreign body penetration into the nose and mouth was more frequently observed in younger children [17], while burns and fractures/dislocations were reported more frequently in older children [17, 18]. Çakmak et al. determined that there is a high correlation between a child having ADHD and a child accidentally drinking corrosive substances [19]. In a birth cohort study with 6,111 children and adolescents, children at eight years of age presenting hyperactivity symptoms were 1.7 times more likely to have already required hospitalization between the ages of zero to seven [4]. The results of systematic reviews show that children with ADHD are have a significant higher risk of accidents [20, 21]. And also this risk is even higher when only poisonings and intoxications are evaluated [22]. The prevalence of accidents in ADHD is, however, thought to not be specific to childhood, and the risk of accidental injury continues into adulthood [23].

Various psychotropic drugs are used in the treatment of ADHD, and treatment response rates vary between studies. Two recent systematic review and meta-analyses have concluded that there is a significant decrease in the risk of unintentional injuries among ADHD children who are treated with ADHD medications [21, 24]. Another meta-analysis also concluded that drug treatment reduced the risk of accidents in children with ADHD [25].

Though many studies have examined the rates of accidents and injuries in children with ADHD, it has been stated that these studies were mostly carried out in the USA and that their results could not be generalized to other cultures [10]. However, studies in Europe [17] and in Turkey also showed that ADHD may make children more vulnerable to accidents [26]. Nevertheless, there is a different limitation to studies already conducted. The majority have examined rates of accidents and injuries in children who were only* diagnosed* with ADHD, but few publications have examined the rates of ADHD in children who are actually admitted to emergency services due to accidents or injuries [26]. Therefore, this research aims to investigate the sociodemographic characteristics of children and adolescents admitted to paediatric emergency services because of accidents or injuries and determine the frequency of ADHD and ODD in these children.

## 2. Method

### 2.1. Sample Population

This study was carried out with children and adolescents who were treated for accidental injuries between September and November 2017 in the Şanlıurfa Mehmet Akif İnan Training and Research Hospital Emergency Department. The participants who applied were primarily diagnosed and treated. Children and parents were informed about the study during an interview, and only patients who agreed to take part in the study and made an affirmation of their agreement have been considered in the research. Parents participating in the study were asked to complete a DSM-IV-based Screening and Assessment Questionnaire (Parent Form) for the children and adolescents, as well as a sociodemographical form, prepared by the authors, which was used to ascertain the parents' sociodemographic characteristics. Only the data of the families who fully filled out the forms were taken into account.

## 3. Material

### 3.1. Socio-Demographic Data Form

For the purpose of the study, characteristics of the child and his/her parents and the economic situation of the family were gathered through the prepared form. Information on the accidents and injuries was filled in by examining the patient's medical file.

### 3.2. Turgay Scale

#### 3.2.1. Screening and Assessment Scale Based on DSM-IV for Behaviour Disorders in Children and Adolescents (Parent Form)

This scale was developed by Turgay for scanning destructive behavioural disorders [27]. Ercan et al. [27] tested the validity and reliability of the scale's Turkish version, developed based on the DSM-IV diagnostic criteria. This version of the scale has been used in many studies before [28, 29]. Although the Turgay scale offers an instructor form, only the parent form was used in our study. The first nine items in the scale evaluated carelessness (DE) and the second nine items evaluated hyperactivity and impulsivity (AH). Appearing together, carelessness, and hyperactivity-impulsivity signified compound-type Attention Deficit Hyperactivity Disorder (ADHD) symptomatology. In the last eight sections, Oppositional Defiant Disorder was (ODD) screened. Each symptom was assessed by a 4-point Likert-type rating scale (0 = None, 1 = A little, 2 = Much, and 3 = Too Much). Each symptom was considered as positive when either the ‘much' or the ‘too much' option was selected.

### 3.3. Statistical Analysis

The data was analysed with SPSS for Windows, version 23.0. Continuous variables in descriptive statistics were expressed as mean and standard deviations; categorical variables were expressed as numbers and percentages. The Mann-Whitney U test was used in analysing data with no normal distribution in binary group comparisons, and the Kruskall-Wallis test was used for comparing more than two groups with no normal distribution. Student's* t*-test was used to compare two groups with normal distribution, and the Anova test compared sets of more than two groups with normal distribution. A chi-square test was used for group comparisons of categorical variables. A value of p < 0.05 was considered statistically significant.

## 4. Results

89 (40.1%) girls and 133 (59.9%) boys diagnosed with soft tissue injuries, burns, fractures, cuts/lacerations, and bleeding between September and November 2017 were included in the study (Total 222). The mean age of children and adolescents was 11.5±3, the youngest being five years old and the oldest 18. The most frequent reason for application was falling off something or somewhere (41.9%), while the most common medical diagnosis, according to the physician evaluation, was soft tissue trauma (41.9%). In this process, 66 (29.7%) children required hospitalisation, while 32 (14.4%) required surgery. The sociodemographic characteristics of children and their families, the reasons for the emergency application, and their medical diagnoses are shown in Table 1.

There was no statistically significant difference between the age, reasons for presentation, and the medical diagnosis of injury in the girls (11.5±3.5) and the boys (11.5±3.2). Hospitalisations after the accident and surgical rates were also similar. However, it was determined that boys had a higher prevalence of psychiatric disorders (P = 0.047) and accident history (P = 0.002) in the past.

Although there were no significant differences in the reasons for emergency service admission and medical diagnoses between the paediatric (5-12 years) and adolescent age groups (13-18 years), the adolescents had higher rates of hospitalisation (P = 0.034).

ADHD symptoms in children were assessed using the DSM-IV-based Screening and Assessment Scale Parent Form for Behavioural Disorders in Children and Adolescents. From the scale filled out by the parents, the average score of inattentiveness was found to be 9.3±7, and the average score of hyperactivity-impulsivity was 10.6±7. The relations between the scale total, subscores, and demographic variables within the sample population are given in Table 2.

**Figure 1:**
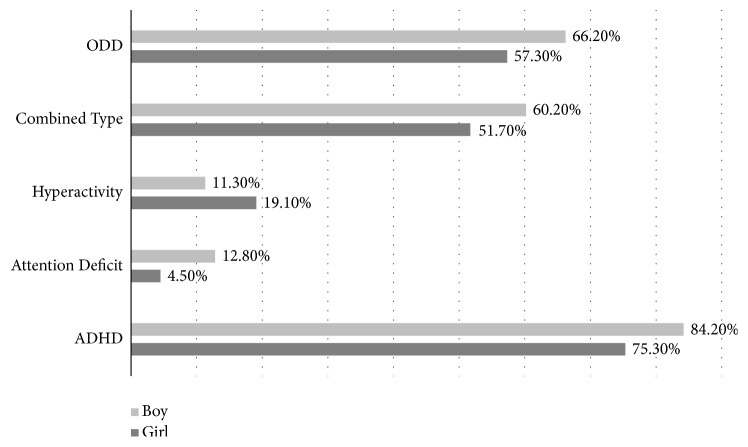
Prevalence of ODD diagnosis and ADHD subtypes according to scale cut-off scores (%). ODD: Oppositional Defiant Disorder and ADHD: Attention Deficit/Hyperactivity Disorder.

Inattentiveness, hyperactivity-impulsivity, and combined ADHD and ODD rates were calculated by accounting for cut-off values that was proposed by the developer of the scale. ADHD symptomatology was observed in 81.6% (179) of children who were admitted to emergency services with accidents and injuries, and ODD symptomatology was observed in 62.6% (139). The behaviour scores of 134 (60.4%) children exceeded the threshold values for both ADHD and ODD. The distribution of ADHD subtypes according to scale cut-off values is given in Figure 1.

The analysis determined that the ADHD subtype in which inattentiveness is dominant was observed more frequently in males (72.9%) than in females (56.2%) (P = 0.010). There was, however, no difference in the distribution of ADHD and subtype diagnoses between female and male patients, nor between child and adolescent groups.

Patients with ADHD (179) and without ADHD (43) were compared with a chi-square test and with* t*-tests; there was no significant difference between the groups in sociodemographic factors, including age and gender, postoperative hospitalization, and surgical necessity.

The analyses also observed that male patients (23%; 7%, *χ*^2^ = 9.815, P = 0.002) and children with a family member who had a psychiatric illness had more accidents (44%; 14%, *χ*^2^ = 9.621, P = 0.002) in the past. When children with both ODD and ADHD symptomatology (n = 134) were compared with children without ODD and ADHD symptomatology (n = 38), it was found that the number of individuals with a psychiatric disorder in the family (100%; 75%, *χ*^2^ = 4.660, P = 0.031) was higher in the comorbidity group. There was no other difference between the groups.

## 5. Discussion

Our study shows that symptomatology of ADHD and ODD is observed in the majority of children who are admitted to emergency service due to accidental injuries. Given that the DSM-IV-based Screening and Assessment Scale used in our study highly predicts clinical ADHD diagnoses, this ratio is significantly higher than the rates established in the community sample of Turkish pupils [27]. These results, therefore, indicate that a significant proportion of children referred to emergency departments due to accidents and injuries have ADHD and ODD and that these rates are well above the population sample. This indicates that these disorders may be effective in children's injuries, suggesting that children admitted to paediatric emergency services due to accidents and injuries should be directed to child psychiatric services after treatment.

Although it is well known that children with ADHD diagnoses are accident-prone and have a risk of injury, most of the studies in this field only investigate the frequency of general accidents and injuries in children with ADHD [26, 30]. The results of our study, differentiated by examining children admitted to emergency departments due to injuries, confirms the suggestion that children with ADHD are at risk of injury. Since ADHD symptoms were seen in 81.6% of children admitted to hospitals due to injuries, ADHD may be confronting children with a high risk of accident. It should also be noted that the symptoms of ODD are quite high among the children in our sample. ODD, which has had a stable incidence rate of 3.7-5.3% in a population sample [7], was found to be significantly higher in our study's sample when compared with the community sample. These results suggest that behavioural problems may be a serious accident predictor for children and adolescents. However, because the evaluation of children was based not on clinical interviews, but on a scale to examine symptoms and the information about children's ADHD symptomatology were not gathered from multiple sources but only from parents and the functionality of children were not included in our study the actual rate of ADHD might be slightly lower than our findings. Future studies which gather information from multiple informants and take functionality of children into account will point ADHD rates more accurately.

Research has reported that ADHD is associated with increased mortality rates in children and adults and that mortality rates are even higher when ADHD is accompanied by ODD or behavioural disorders [14]. The high prevalence of ADHD and ODD comorbidities in our sample suggests that being accident-prone may be associated with increased mortality in children with ADHD. Similarly, accident risks have been reportedly higher in children with ADHD and co-morbid ODD [16, 31]. The presence of high ADHD and ODD symptoms in our sample of children supports this view. However, it should be noted that the high ADHD-ODD codiagnosis is not unique to our study sample and that similar co-morbidities are reported in community sample studies [7].

In a similar study, ADHD frequency was reported to be 28% in children who were admitted to emergency services due to injuries, and the likelihood of ADHD was three times higher in children who were admitted to the hospital for injuries than in those who were admitted for appendicitis [30]. In a study conducted in Turkey, ADHD symptomatology was also found to be more common in children who were hospitalized for extremity fractures due to accidents [32]. Although our results also indicate increased rates of ADHD in children with unintentional injuries, the prevalence of ADHD was higher in our sample than in previous studies. Gathering information from only one source through a questionnaire and not including functionality of children in evaluation of children might lead to getting higher rates of ADHD in our sample. Cultural differences may also have a role in different rates between similar studies across different countries which means that Turkish children with ADHD might be more prone to accidents than non-Turkish children. However, further studies are needed to conclude whether accident proneness is different in children with ADHD in different cultures.

Lange et al. conducted a comparable study in Germany, with a sample representative of western European society and stated that ADHD increases the risk of accidents, but electing to receive or not receive treatment may not affect the risk of accidents [10]. Most studies investigating these risks in ADHD have shown that individuals with ADHD experienced an increased risk of accidents, evidenced by their answers to questions asking about accidents and injuries they experienced in the past [26]. Since those studies relied on participants' autobiographical memories, it is possible that their results may suffer from a disremember bias [30]. The data in our current study was made of information obtained from the children's parents after the accidents. Thus, we attempted to circumvent disremember bias and determine how many of the accident sufferers had ADHD symptoms. Although it is expected that the parents, in the postaccident situation, will be emotionally affected, we did not observe that this caused the families to fill in the scales biasedly. However lack of a control group and a quantitative measurement tool to assess the severity of the injury and performing the study under emergency service conditions reduces the impact of our findings.

Since such results indicate that ADHD symptoms are common in children admitted to emergency services for accidents and injuries [18, 25], publications show that children with ADHD are at an increased risk of accidents [10, 19, 33], and reports suggest that proper ADHD treatment can reduce accident risks, it is vital that doctors in emergency services define ADHD symptomatology and refer children (those admitted for accidental injuries and those with recurrent accidents) to child psychiatric services. Such action can possibly prevent future accidents and injuries by treating the cause (ADHD), not just the effect (the injury).

ADHD has generally been observed more frequently in males, and the ratio of male to female is about 2:1 [5]. It is, therefore, surprising that, in our study, there was no gender difference, in either cumulative or ADHD subtypes, except for the carelessness subtype. This conclusion suggests that ADHD may cause accidents and injuries in girls more often than previously understood or that having ADHD may make girls more vulnerable to accidents. Interestingly, in a study evaluating 1.92 million people, the mortality rates of girls with ADHD were also found to be higher than the mortality rates of boys with ADHD [14]. Although it is not one of the main objectives of our study design, we consider this finding to be important, and, in later studies, it will be necessary to examine whether ADHD increases the risk of accident more in girls than boys.

Beyond being merely an accident-prone individual, it has been stated that injuries to children and adolescents with ADHD are more severe; hospitalisations after the accident are longer; and disability rates are higher [33]. Although we did not have quantitative tools to measure the level of injury in our study, it is plausible that being hospitalised and undergoing surgery could roughly reflect the severity of the injury. Thus, to compare the severity of injuries in children with and without ADHD symptomatology, we checked those parameters, and the results of our comparison showed that there was no significant difference between children with and without ADHD. This seems to contradict previous findings, which claim that, in children with ADHD, accidents and injuries are likely to be more severe [34]. However, because we lacked a quantitative tool to measure the severity of injury and only looked at whether the children were admitted rather than the duration of hospitalisation, it is impossible for us to make a clear comment on this issue, which limits the effectiveness of our findings.

It has been reported that boys are exposed to accidents and injuries more often than girls [35]. The data of our study also supported this finding, and it was observed that most of the patients were male. The most common cause of injury among children in our sample was falling off something, and this was followed by crash/wrenching and burns. Literature in the field, including a related study in Turkey, corroborates our sample, also showing that falling off something is the most common cause of injury [34]. Another Turkish study observed that the most common cause of injury was falling off something, which was followed by traffic accidents and burns [35].

In some publications, it has been stated that the types of injuries may vary according to age [17, 18], but our study contradicts this literature because there was no significant difference in terms of emergency admission and medical diagnoses between child and adolescent age groups. However, this discrepancy could exist because the causes of the injuries we have worked with were not elaborated. Our study also found that all scale scores of children who have a family member with a psychiatric disorder were significantly higher than those without such a family member and that these children had frequent accident history in their pasts. The presence of an individual with a psychiatric disorder in a child's family was described as an adverse childhood experience [31], and this was also considered a risk factor for children's psychological and medical health [31]. Our study supported this assertion, determining that the presence of someone with a psychiatric disorder in the family is related to common ADHD symptoms and previous accident history. This risk factor may be caused by similar genetic structures between the affected children and the family members with psychiatric disorders, impaired family functioning, or the complex interactions of both.

## 6. Conclusion

Mortality is reportedly increased in children with ADHD, and this correlates with increased accident rates in those children [14]. Our study is also important because it shows that adverse effects of ADHD are not only limited to academic and social functioning but may also be related to an increased risk of accidents and injuries in children and adolescents. If physicians and assistant health personnel working in emergency traumatology departments refer patients and adolescents, who have experienced accidents and injuries, to child and adolescent psychiatric services after treatment, then accidents, injuries, and related death and disabilities may be prevented.

## Figures and Tables

**Table 1:** Sociodemographic variables and accident/injury data.

	**n**	%
Children (5-12 ages)	141	63.5%
Male (13-18 ages)	81	36.5%
**Going to school?**	208	93.7%
**Monthly income**		
0-1000 TL	38	17.1%
1000-2000 TL	60	27.0%
2000-3000 TL	59	26.6%
3000-4000 TL	37	16.7%
7000 TL and more	28	12.7%
**Kinship between parents**	106	47.7%
**Family status**		
Together	207	93.2%
Separated	5	2.3%
Divorced	8	3.6%
Died	2	0.9%
**Number of siblings**		
1	20	9.0%
2	45	20.3%
3	64	28.8%
4 and more	93	41.9%
**Presence of an individual living at home other than family**	20	9.0%
**Family history of psychiatric illness**	16	7.2%
**Presence of former psychiatric illness**	10	4.5%
**Previous accident or surgery story**	36	16.2%
**Presence of any disease**	24	10.8%
**The reason for the emergency service application**		
Falling off	124	55.9%
Burn	25	11.3%
Traffic accident	15	6.8%
Sharp object injury	2	0.9%
Crush or wrenching	56	25.2%
**Diagnosis at emergency service**		
Soft tissue trauma	93	41.9%
Fracture	72	32.4%
Burn	25	11.3%
Cut-laceration	27	12.2%
Bleeding	5	2.3%
**Surgery after trauma**	32	14.4%
**Hospitalization after trauma**	66	29.7%

**Table 2:** Relation between scale scores and demographic variables.

	**Carelessness**	**Hyperactivity and Impulsivity**	**Complex type ADHD**	**ODD**
	*Score (Mean±SD)*	*Score (Mean±SD)*	*Score (Mean±SD)*	*Score (Mean±SD)*
**All patients**	9.3±6.9	10.6±7.3	19.9±12.7	7.7±5.7
**Gender (n)**				
Male (133)	10±6.8	10.5±7.5	20.5±12.8	7.8±5.5
Female (89)	8.3±6.9	10.8±7.1	19.1±12.6	7.6±6
**p**	0,085	0.795	0.436	0.778
**Age group (n)**				
Paediatric (141)	9.6±7.1	11.5±7.5	21.2±13.1	7.9±5.9
Adolescent (81)	8.8±6.5	9±6.6	17.8±11.9	7.4±5.3
**p**	0.374	**0.013** **∗**	0.056	0.541
**Presence of former psychiatric illness**				
Yes	13.6±7.3	13.5±10.5	27.1±14.7	11.3±4.2
No	9.1±6.8	10.5±7.2	19.6±12.6	7.5±5.7
**p∗**	**0.044**	0.201	0.068	**0.041**
**Previous accident history**				
Yes	12.0±8.1	11.4±8.9	23.4±16.4	8.7±5.3
No	8.8±6.4	10.4±6.9	19.2±11.8	7.5±5.7
**p∗**	**0.009**	0.469	0.070	0.234
**Presence of an individual living at home other than family**				
Yes	12.3,±7.8	13.6±8.0	26.0±14.8	8.9±5.6
No	9.0±6.7	10.3±7.2	19.4±12.4	7.6±5.7
**p∗**	**0.040**	0.053	**0.027**	0.341
**Psychiatric disease in family**				
Yes	16.2±8.5	16.1±7.4	32.3±15.0	12.7±5.1
No	8.8±6.4	10.2±7.1	18.9±12.6	7.3±5.5
**p∗**	**<0.001**	**0.002**	**<0.001**	**<0.001**

*∗t-*test p < 0.05.

## Data Availability

The data used to support the findings of this study are available from the corresponding author upon request.
